# Implementation of patient charges at primary care facilities in Kenya: implications of low adherence to user fee policy for users and facility revenue

**DOI:** 10.1093/heapol/czu026

**Published:** 2014-05-16

**Authors:** Antony Opwora, Evelyn Waweru, Mitsuru Toda, Abdisalan Noor, Tansy Edwards, Greg Fegan, Sassy Molyneux, Catherine Goodman

**Affiliations:** ^1^Kenya Medical Research Institute - Wellcome Trust Research Programme, P.O. Box 43640, Nairobi, Kenya, ^2^Kenya Medical Research Institute - Wellcome Trust Research Programme, P.O. Box 230, Kilifi, Kenya, ^3^Malaria Public Health and Epidemiology Group, Centre for Geographic Medicine, Kenya Medical Research Institute - Wellcome Trust Research Programme, P.O. Box 43640, Nairobi, Kenya, ^4^Centre for Tropical Medicine, Nuffield Department of Clinical Medicine, University of Oxford, Centre for Clinical Vaccinology and Tropical Medicine, Oxford OX3 7LJ, UK, ^5^MRC Tropical Epidemiology Group, London School of Hygiene & Tropical Medicine, Keppel St., London WC1E 7HT, UK and ^6^Department for Global Health and Development, London School of Hygiene & Tropical Medicine, Keppel St., London WC1E 7HT, UK

**Keywords:** Health-care financing, user fees, primary health care, Kenya

## Abstract

With user fees now seen as a major hindrance to universal health coverage, many countries have introduced fee reduction or elimination policies, but there is growing evidence that adherence to reduced fees is often highly imperfect. In 2004, Kenya adopted a reduced and uniform user fee policy providing fee exemptions to many groups. We present data on user fee implementation, revenue and expenditure from a nationally representative survey of Kenyan primary health facilities. Data were collected from 248 randomly selected public health centres and dispensaries in 2010, comprising an interview with the health worker in charge, exit interviews with curative outpatients, and a financial record review. Adherence to user fee policy was assessed for eight tracer conditions based on health worker reports, and patients were asked about actual amounts paid. No facilities adhered fully to the user fee policy across all eight tracers, with adherence ranging from 62.2% for an adult with tuberculosis to 4.2% for an adult with malaria. Three quarters of exit interviewees had paid some fees, with a median payment of US dollars (USD) 0.39, and a quarter of interviewees were required to purchase additional medical supplies at a later stage from a private drug retailer. No consistent pattern of association was identified between facility characteristics and policy adherence. User fee revenues accounted for almost all facility cash income, with average revenue of USD 683 per facility per year. Fee revenue was mainly used to cover support staff, non-drug supplies and travel allowances. Adherence to user fee policy was very low, leading to concerns about the impact on access and the financial burden on households. However, the potential to ensure adherence was constrained by the facilities’ need for revenue to cover basic operating costs, highlighting the need for alternative funding strategies for peripheral health facilities.

**KEY MESSAGES**

- In 2004 Kenya introduced a reduced and uniform user fee policy providing fee exemptions to many groups.
- Adherence to the policy is very low with most patients paying more than the specified amount, very few receiving waivers, and a quarter required to purchase additional supplies from private shops.
- User fee revenue represents almost all the cash income of facilities, and is used to cover basic operating costs such as support staff, supplies and travel allowances.
- Attempts to enhance adherence to the user fee policy and to further reduce official charges should be accompanied by strategies to compensate facilities for lost revenue and carefully monitor fees charged.

## Introduction

User fees have been widely used as a source of health facility financing in the developing world (Ridde and Morestin 2011). In many African countries, fees were introduced in the 1980s with the aim of raising additional funds and curbing frivolous demand for health services (UNICEF 1989–1993). Through waivers and exemptions, it was hoped that the poor and specific categories of patients such as young children and pregnant women would be protected from costs (Bitran and Giedion 2003; McPake et al. 1992). However, several decades later, many studies have shown that these aims have not been achieved. User fees have reduced demand for health services, especially among the poor, many countries have struggled to identify the poor for waivers, and expected improvements in quality of care have rarely materialized (Lagarde and Palmer 2008; McPake et al. 2011; Ridde and Morestin 2011). Moreover, user fees have been found to be inefficient in raising substantial revenues for health facilities (James et al. 2006).

With user fees now seen as a major hindrance to universal health coverage, there are increasing calls from the World Health Organization (WHO), the United Nations Children's Fund (UNICEF) and other organizations for countries to abolish or reduce fees (Evans et al. 2010; James et al. 2006; Save the Children 2005; Yates 2009). Many countries have introduced fee reduction or elimination policies, including implementing new exemptions for particular patient groups or health conditions (for example in Kenya, Niger, Mali, Ghana, Nigeria, Burkina Faso, Tanzania and Zambia), or removing fees across the board as in Uganda and South Africa (Gilson 1997; McPake et al. 2011; Meessen et al. 2011a, 2011b; Ridde and Morestin 2011). Although the goal is to reduce costs for patients and increase access to health care, there is growing evidence that adherence to reduced fees is often highly imperfect. Fees are charged, either formally or informally, for care that should be free, and where fees are set at a particular level, charges in practice are often higher (Chuma et al. 2009; Lewis 2007; Meuwissen 2002; Opwora et al. 2011).

In 2004, Kenya adopted a reduced and uniform user fee policy—the ‘10/20 policy’. The policy aimed to effect a change from high and variable fees to standardized fees at a flat rate of Kenyan Shillings (KES) 10 (USD 0.13^1^) in dispensaries and KES 20 (USD 0.26) in health centres. Dispensaries are the lowest level of outpatient health facility in Kenya, while health centres are slightly larger and may have some inpatient beds. Full fee exemptions were to be provided for specific services including treatment for malaria, tuberculosis (TB) and sexually transmitted diseases, all care for under 5 year olds, deliveries, and antenatal care (ANC). Waivers were also to be provided to patients from particularly poor households. The guidelines were unclear on whether laboratory fees should be included in these fee levels. User fee income was generally to be used locally for operational and maintenance costs, and managed by local health facility committees made up of the health worker in-charge of the facility (known as the in-charge) and local residents (Molyneux et al. 2012). The government continued to provide facilities with infrastructure, trained health workers, drug kits and medical supplies. The 10/20 policy had the potential to expand access to health care through increasing affordability, but also to lead to a reduction in revenues available for day-to-day operations at the health facility level. A study conducted in two districts three years after implementation of the policy suggested that the latter concern contributed to imperfect adherence to the policy by health facility staff (Chuma et al. 2009).

In this article, we present data on user fee implementation, revenues and expenditure from a nationally representative survey of public primary health facilities in Kenya. We assess adherence to the user fee reduction policy as reported by in-charges, and actual fees facility users report paying. We then explore potential reasons for poor adherence to the official policy by assessing associations between adherence and facility characteristics; and draw on income and expenditure data to outline the role of user fees in facility revenue and activities.

## Methods

We conducted a nationally representative survey of public primary care facilities across all eight Kenyan provinces. We followed a two stage sampling process. First, we randomly selected three districts per province in seven provinces (excluding Nairobi; *n* = 21 districts), and one district from each of the three municipal areas (Nairobi, Mombasa and Kisumu).^2^ Within each of the 24 selected districts our sampling frame included all government-owned health centres and dispensaries staffed by at least one qualified nurse, or—in exceptional cases—considered by the government as adequately supervised by qualified staff. Next, we stratified the sample by facility type (health centre and dispensary), and for each facility type randomly selected seven facilities per district. In districts with less than eight facilities of a given type, we surveyed all relevant facilities. The sample size was based on the needs of a before and after evaluation of a new health financing mechanism, for which this survey represented the baseline.

Data were collected from 248 facilities between July and September 2010. Of these, 209 facilities were in non-municipal areas and 39 in municipal areas.^3^ At each facility we conducted a structured survey, comprising an interview with the facility in-charge, exit interviews with three outpatients seeking curative care (or their caretaker), and a records review on facility income, expenditure and utilization. The interview with the in-charge covered facility management structures, staffing, sources of income, and user fees charged for specified types of patients. Patients/caretakers were considered eligible for interview if they were aged 16 years or over, had come to the facility for treatment, normally resided in the local area, had lived there for at least 6 months, and if this was their nearest government health facility. Exit interviews with outpatients seeking curative services covered user fees paid and owed to the facility for all services received that day, and whether exit interviewees were required to buy extra supplies to complete treatment. Records on facility income, expenditure and utilization were reviewed, with data included in the analysis for those facilities with at least 8 months of valid records between July 2009 and June 2010. For these facilities, figures for the missing months were imputed using the median for that facility for the months with records.

Interview data and records were entered at the point of data collection in Microsoft (MS) Access forms on mini-laptops. These were then checked every day by team supervisors for consistency and accuracy before being transferred by email to a central data manager, who communicated any queries back to the field teams. Analysis was conducted in Stata version 11 (Stata Corp, College Staion, TX, USA).

The analysis incorporated the survey design by adjusting for clustering at the district and facility levels, stratified by facility type and municipal/non-municipal area. Differences in sampling probability across facility type and districts were accounted for by using sampling weights.

### Measuring adherence to the user fee policy

To measure adherence to the national user fee policy, tracer conditions were selected to represent a mix of cases commonly treated at Kenyan primary health facilities, and the government’s priority public health interventions across different age groups, genders and illness types. Tracers selected that should be exempt from user fees were: child with malaria, adult with TB, ANC client (first visit), child with pneumonia, adult with malaria, adult with gonorrhoea, and delivery services. The tracer selected that required a payment of KES 10 or 20 at a dispensary or health centre respectively was an adult with pneumonia.

Adherence was measured from facility in-charge responses regarding fees normally charged at the facility for patients presenting with the tracer conditions, both including and excluding laboratory fees given their ambiguous status within the 10/20 policy. Costs of purchasing a patient record card were not included as these tend to be one-off payments only required on the first visit to that facility. Facilities were recorded as non-adherent to a tracer if in-charges reported charging any fees to clients who should be exempt or charging more than the stipulated amount for an adult with pneumonia. The proportion of facilities adhering to the user fee policy for each tracer condition and overall adherence (i.e. adherence to all tracer conditions) were computed.

In order to explore factors associated with adherence, three tracer conditions were selected that were applicable to all facilities, but found to have varying adherence to user fee policy: child with malaria, adult with TB, and ANC client. Variables hypothesized to be positively associated with adherence included those related to location (municipal facilities, less remote facilities, facilities in less poor locations), recent supervision by the District Health Management Team, recent meeting of the health facility committee, and facilities displaying official user fees. These variables were selected on the basis that these facilities might be considered to be more accessible to health-care managers, and their users might be better informed about national user fee policy, and more demanding of their rights to exemptions. In addition, health centres were hypothesized to be likely to charge more than dispensaries to ensure income for their more complex services and larger numbers of support staff; and facilities with laboratory services to charge more because these services often act as an income generation activity. Association with user fee policy adherence was assessed using the Pearson chi-square (χ^2^) test.

Facility remoteness was calculated as the distance between the sampled facility and the nearest of the 268 main towns (as defined in the 2009 Kenyan national census), measured using a straight-line method in ArcGIS 9.2 (ESRI Inc., USA). Facilities were categorized into near (0–5 km), middle (6–30 km), and far (>30 km) from main towns. To measure the poverty level of the facility’s local area, we used the proportion of the population above the poverty line in the location (second lowest administrative area) in which the sampled facility was located. Methods for the calculation of the poverty level by location are presented elsewhere (Toda et al. 2012). Sampled facilities were grouped into weighted socio-economic status (SES) quintiles.

This study was approved by the Kenya Medical Research Institute (KEMRI) Ethics Review Committee and the London School of Hygiene and Tropical Medicine in the UK. Informed consent was obtained verbally for all interviews.

## Results

### Characteristics of facility in-charges and exit interviewees

Interviews were conducted with 248 facility in-charges and 698 facility clients (Table 1). About three quarters of in-charges (73.1%) were aged 25–44 years, and a quarter (26.5%) aged 45 years or above. About half of in-charges (47.9%) were female, although this proportion was higher in municipal areas, at 75.3% and 66.7% in dispensaries and health centres, respectively. Most in-charges were qualified health workers, most often enrolled nurses (47.8%), registered nurses (29.5%) and clinical officers (13%), the remainder being community health workers (5.3%) or having other health qualifications (4.5%), such as retired nurse, laboratory technologist/technician, and public health technician.

**Table 1.** Summary of data collected

	Non-municipal		Municipal		Total
	Dispensaries	Health centres	Dispensaries	Health centres	
In-charge questionnaire	144	65	21	18	248
Exit interview questionnaire	400	192	53	53	698^a^
Document review tool	140	65	21	18	244

^a^753 patients were approached; 50 declined to be interviewed, 3 did not meet the inclusion criteria, and 2 were later excluded because they were unable to answer the questions consistently.

Slightly over half (56.7%) of exit interviewees were seeking curative health services for themselves, with the remainder seeking care for sick children. Just over half of exit interviewees were aged 25–44 years (54.5%), and had completed primary school (56.5%). Almost two thirds (64.9%) were female, although in municipal dispensaries less than half (47.4%) were female.

### Facility adherence to user fee policy—in-charge reports

**Figure 1:**
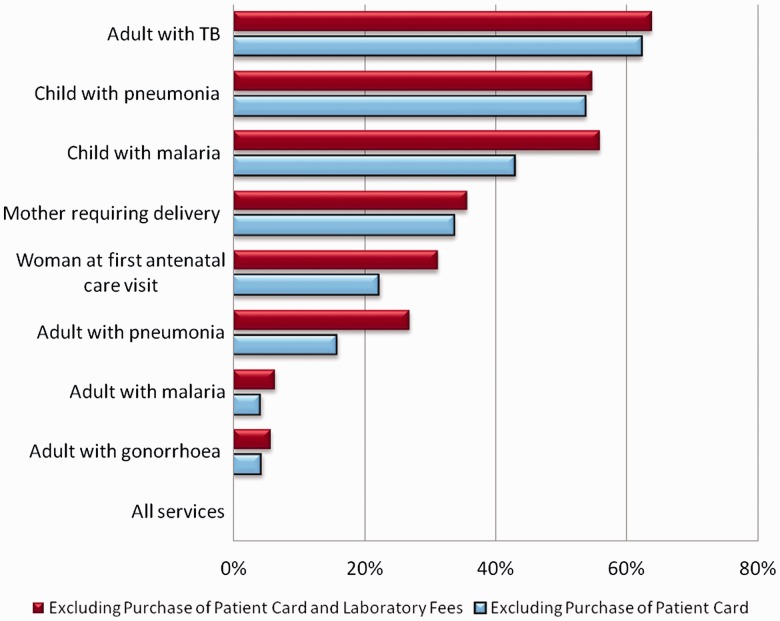
Percentage of facilities adhering to user fee policy (*N* = 248). *Source:* In-charge interviews.

When adherence was assessed across all eight tracer conditions combined, including laboratory fees, none of the facilities adhered fully to the official user fee policy (Figure 1). When the services were analyzed individually, adherence was highest for an adult with tuberculosis (62.2%), followed by a child with pneumonia (53.7%). Adherence was lowest for an adult with malaria (4.2%) and an adult with gonorrhoea (4.3%). When laboratory fees were excluded from stated user fees, we still found that no facilities reported adhering to the policy across all tracers, with similar patterns of adherence across age and illness groups. The degree to which patients were over-charged (including laboratory fees) varied from a median of zero for an adult with TB and a child with pneumonia, to KES 40 (USD 0.53) for an adult with malaria and KES 50 (USD 0.66) for an adult with gonorrhoea (Table 2). Particularly high levels of over-charging were reported in non-municipal health centres for first ANC visit and delivery [medians of KES 120 (USD 1.58) and KES 150 (USD 1.97), respectively].

**Table 2.** Median value of over-charge^a^ reported compared to user fee policy (USD) (excluding purchase of patient card)

	Non-municipal		Municipal		Total
	Dispensaries	Health centres	Dispensaries	Health centres	
*N*	144	65	21	18	248
	median (IQR)	median (IQR)	median (IQR)	median (IQR)	median (IQR)
Child with malaria	0.26 (0–0.39)	0 (0–0.39)	0 (0–0.39)	0.59 (0–1.31)	0.13 (0–0.39)
Adult with malaria	0.39 (0.26–0.66)	0.66 (0.26–0.92)	0.39 (0.26–0.66)	1.05 (0.26–3.81)	0.53 (0.26–0.66)
Child with pneumonia	0.13 (0–0.26)	0 (0–0.13)	0 (0–0.26)	0.39 (0–1.31)	0 (0–0.26)
Adult with pneumonia	0.13 (0–0.53)	0 (0–0.39)	0.13 (0–0.53)	0.33 (0–2.1)	0.13 (0–0.53)
Adult with TB	0 (0–0.13)	0 (0–0.26)	0 (0–0)	0 (0–0.26)	0 (0–0.13)
Adult with gonorrhoea	0.53 (0.26–0.92)	0.92 (0.39–1.51)	0.66 (0.26–1.18)	0.46 (0.26–1.97)	0.66 (0.26–1.05)
***N***	**137**	**64**	**19**	**18**	**238**
Woman at first antenatal care visit	0.26 (0.13–1.31)	1.58 (0.26–2.76)	0.26 (0–0.66)	1.18 (0.26–2.63)	0.26 (0.13–1.97)
***N***	**111**	**57**	**15**	**12**	**195**
Mother requiring delivery	0.13 (0–0.66)	1.97 (0.26–3.94)	0 (0–0.26)	0.13 (0–3.41)	0.26 (0–1.97)

*Source:* In-charge interviews.

*Note:* Data were missing for two facilities for “child with malaria”, one facility for “adult with malaria”, three facilities for “child with pneumonia”, one facility for “adult with pneumonia”, 16 facilities for “adult with TB”, and five facilities for “adult with gonorrhoea”.

^a^Over-charge is the amount charged minus amount that should be charged, which for all tracers except “adult with pneumonia” is zero. For “adult with pneumonia” figures include data for 11 facilities which reported no charge.

Most facilities (90.4%) reported giving waivers on the basis of poverty (Table 3). Of those providing waivers, a median of 15 people were waived in the preceding quarter, representing only about 1% of the median outpatient curative visits. The median amount waived in the preceding quarter per facility was USD 7.22.

**Table 3:** User fee waivers

	Non-municipal		Municipal		Total
	Dispensaries	Health centres	Dispensaries	Health centres	
*N*	144	65	21	18	248
	% [95% CI]	% [95% CI]	% [95% CI]	% [95% CI]	% [95% CI]
**Facility in-charge reported that waivers were given on the basis of poverty**	88.7 [83.4–92.4]	100	84.9 [21.1–99.2]	100	90.4 [86.2–93.4]
**Of those that give waivers (*N* = 230), median number of people waived and value of waivers in the last quarter (April 1st and June 30th, 2010)**					
*N*	130	64	18	18	230
	Median (IQR)	Median (IQR)	Median (IQR)	Median (IQR)	Median (IQR)
Number of people waived	15 (5–57)	10 (5–30)	10 (6–30)	3 (0–57)	15 (5–51)
Amount waived (USD)	7.22 (1.31–26.25)	7.61 (1.31–13.13)	7.09 (5.25–13.91)	3.94 (0–14.96)	7.22 (1.84–24.42)

*Source:* In-charge interviews.

*Note*: Data were not available for one facility for whether waivers were given, for 105 facilities for number of people waived, and for 107 facilities for the amount waived.

### User payments - reports of exit interviewees

Clients attending curative services were asked if they had paid any money for services received that day and if so, how much. Three quarters of exit interviewees (74.7%) had paid some money (Table 4). The median amount paid by patients irrespective of age was KES 30 (USD 0.39). In addition, 5% of interviewees owed the facility some additional money for the treatment they had received that day, with a median debt in those aged 5 years and above of KES 20 (USD 0.26), and for those aged under 5 of KES 25 (USD 0.33). Furthermore, a quarter of interviewees were required to purchase additional medical supplies at a later stage from a private drug retailer, in most cases medicines (91.9% of those requiring additional purchases), but also injection needles, syringes, and bandages.

**Table 4.** Payments for health services reported by patients exiting the health facility

	Non-municipal		Municipal		Total
	Dispensaries	Health centres	Dispensaries	Health centres	

*N*	400	192	53	53	698
	% [95% CI]	% [95% CI]	% [95% CI]	% [95% CI]	% [95% CI]
Paid for services received today	76.8 [68.7–83.3]	68.3 [57.8–77.1]	60.1 [22.2–88.8]	67.2 [32.1–89.9]	74.7 [68.7–80.0]

Of those that paid for services received today, median amount paid (USD):					
	Median (IQR)	Median (IQR)	Median (IQR)	Median (IQR)	Median (IQR)
*N*	221	112	25	25	383
Patients 5 years and over	0.39 (0.26–0.66)	0.53 (0.26–0.92)	0.92 (0.26–1.31)	0.26 (0.26–1.31)	0.39 (0.13–0.66)
*N*	68	27	8	10	113
Patients under 5 years	0.39 (0.26–0.66)	0.39 (0.26–0.66)	0.53 (0.26–1.58)	1.31 (0.39–3.15)	0.39 (0.26–0.66)

Of all patients					
*N*	398	192	53	53	696
	% [95% CI]	% [95% CI]	% [95% CI]	% [95% CI]	% [95% CI]
Patient still owes facility money for services received today	5.9 [3.1–11.1]	1.4 [0.3–5.7]	0	5.6 [3.0–10.3]	5.0 [2.6–9.5]
Patient needs to buy drugs or other supplies elsewhere	21.7 [14.5–31.1]	36.2 [27.1–46.4]	40.0 [15.9–70.3]	35.6 [25.9–46.7]	24.9 [18.4–32.7]

*Source:* Exit interviews.

### Factors affecting user fee policy adherence

Table 5 shows the prevalence of key facility characteristics hypothesized a priori to affect user fee adherence, as described above. The table also shows the association of these characteristics with adherence to user fees (including laboratory fees) for the three selected tracer conditions: child with malaria, adult with tuberculosis, and woman attending first antenatal clinic. No consistent patterns of associations were identified across tracers, with non-adherence common in facilities with a wide range of characteristics. The only statistically significant findings were that municipal facilities were more likely to adhere to free care for TB than non-municipal ones; that health centres were more likely than dispensaries to adhere for a child with malaria, but dispensaries were more likely to adhere for ANC; and that facilities without laboratories were more likely to adhere for ANC. If laboratory fees are excluded, again there are no consistent patterns of association; the associations for ANC fees with facility type and laboratory services are no longer significant, but adherence is more likely for a child with malaria in a facility with a laboratory (data not shown).

**Table 5.** Factors associated with adherence to user fee policy for three tracer conditions^a^^,^^b^

		Child with malaria % [95% CI]	Adult with TB % [95% CI]	Woman at first ANC visit % [95% CI]
Area type	*N*	246	232	238
	Non-municipal	42.4 [33.8,51.4]	61.2 [50.4,71.0]	22.7 [10.0,43.5]
	Municipal	55.1 [22.9,83.6]	80.8 [70.8,88.0]	29.2 [12.7,53.8]
		***P* = 0.4660**	*P* = 0.0074*	***P* = 0.6158**
Type of facility	*N*	246	232	238
	Dispensary	40.8 [32.3,49.8]	63.3 [51.0,74.1]	25.3 [11.3,47.5]
	Health centre	54.0 [43.1,64.5]	57.1 [46.6,67.0]	11.3 [4.4,25.9]
		*P* = 0.0056*	***P* = 0.3790**	*P* = 0.0332*
% Households living above the poverty line (quintile)	*N*	246	232	238
	Least poor	43.1 [26.1,61.9]	54.7 [37.8,70.5]	38.0 [23.7,54.7]
	4th	40.9 [19.9,66.0]	73.4 [62.0,82.3]	32.3 [12.2,62.2]
	3rd	21.3 [5.3,56.6]	61.4 [34.2,83.0]	11.1 [3.5,30.3]
	2nd	42.3 [26.9,59.4]	66.4 [49.2,80.2]	19.0 [3.0,64.4]
	Poorest	69.8 [50.5,83.9]	53.4 [30.4,75.1]	12.9 [3.3,39.0]
		***P* = 0.1282**	***P* = 0.4022**	***P* = 0.1934**
Distance from main town (km)	*N*	246	232	238
	Near (0–5 km)	38.4 [21.3,58.9]	60.2 [47.6,71.6]	10.1 [3.8,24.0]
	Middle (6–30 km)	41.0 [32.2,50.4]	63.0 [49.1,75.0]	25.3 [12.0,45.7]
	Far (>31 km)	55.7 [33.7,75.7]	62.2 [42.6,78.5]	32.2 [6.2,77.3]
		***P* = 0.3830**	***P* = 0.9371**	***P* = 0.2394**
Supervision in the last quarter	***N***	232	220	226
	No	42.7 [31.9,54.3]	64.4 [50.9,75.9]	21.6 [8.0,46.7]
	Yes	40.9 [29.2,53.7]	58.6 [44.0,71.8]	23.9 [11.2,44.0]
		***P* = 0.8175**	***P* = 0.4415**	***P* = 0.7790**
Full meeting of health facility committee in the last quarter	*N*	246	232	238
	No	45.3 [29.6,62.0]	61.1 [44.9,75.1]	19.4 [5.8,48.5]
	Yes	42.2 [32.0,53.2]	62.5 [49.4,74.0]	24.0 [11.6,43.2]
		***P* = 0.7646**	***P* = 0.8859**	***P* = 0.5673**
Official user fees displayed and visible to users	*N*	241	227	233
	No	48.3 [35.7,61.1]	66.4 [58.6,73.5]	22.4 [9.6,44.0]
	Yes	36.9 [26.0,49.2]	64.2 [46.2,78.9]	30.4 [12.5,57.2]
		***P* = 0.1514**	**P = 0.8071**	***P* = 0.5197**
Laboratory services available	*N*	244	230	236
	No	47.5 [35.1,60.3]	69.3 [55.7,80.2]	32.1 [14.1,57.6]
	Yes	33.1 [24.0,43.6]	54.9 [39.6,69.4]	8.4 [2.6,24.0]
		***P* = 0.1028**	***P* = 0.0738**	*P* = 0.0057*

*Source:* In-charge interviews.

^a^Adherence to user fee policy described in this table is based on the official fees (user fees which should be charged for tracer cases according to official policy).

^b^The results presented in this table include fees for laboratory services, but exclude costs for patient cards which are not required at all facilities, and are usually only required for the first visit.

*Statistically significant at the 0.05 level.

### Role of user fees in facility income and expenditure

To understand the role of user fee income, we reviewed facility records on income and expenditure for the year July 2009 to June 2010 to calculate the proportion of income from user fees, and what that user fee money supports. Just under three-quarters of facilities had data available on income (74.2%) and expenditure (73.6%) for at least 8 months out of 12 (Table 6). Roughly half of the dispensaries (42.9%) and health centres (50.0%) in municipal areas had sufficient expenditure records available, compared to 73.9% of dispensaries and 80.7% of health centres in non-municipalities.

**Table 6.** Facilities with income and expenditure data available for at least 8 of the 12 months between July 2009 and June 2010

	Non-municipal		Municipal		Total
	Dispensaries	Health centres	Dispensaries	Health centres	
*N*	140	65	21	18	244
	% [95% CI]	% [95% CI]	% [95% CI]	% [95% CI]	% [95% CI]
Income	75.8 [63.1–85.1]	67.9 [56.5–77.5]	72.4 [28.5–94.5]	64.3 [25.9–90.3]	74.2 [64.5–82.1]
Expenditure	73.9 [61.1–83.6]	80.7 [60.0–92.1]	42.9 [11.5–81.2]	50.0 [11.6–88.4]	73.6 [64.6–81.0]

*Source:* Facility Records Review.

Some form of cash income was received by 82.0% of facilities, with a median annual income of US$ 683 [inter-quartile range (IQR) US$ 115–2092]. Median income was highest in municipal health centres (US$ 4344), followed by non-municipal health centres (US$ 1823), and non-municipal dispensaries (US$ 641), and lowest in municipal dispensaries (US$ 453).

Nearly all income received was from user fees, with just a couple of facilities receiving income from selling insecticide treated bednets (0.9%). Other sources of income included occasional donations from various organizations, and income generating activities, the most common being running a laboratory. Some facilities also engaged in farming, among other investments.

Most facility in-charges reported that user fee income was retained at the facility (91.7% of facilities), but this overall figure masks marked variation between non-municipalities and municipalities. In municipalities a high proportion of facilities remitted income to either the Nairobi Health Management Board or elsewhere, with only 35.4% of municipal health centres and 33.3% of municipal dispensaries retaining user fee revenues at the facility level.

**Figure 2:**
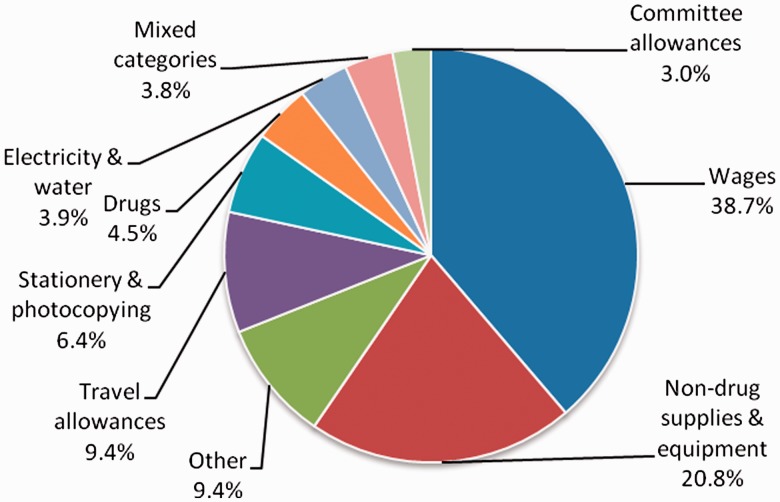
Facility level expenditure by category [July 2009–June 2010] (includes expenditure of facility revenue from all sources). *Source:* Facility records reviews.

Figure 2 shows the breakdown of total facility expenditure by category for the financial year July 2009 to June 2010. Over a third (38.7%) of all facility expenditure was on wages, 20.8% on non-drug supplies and equipment, and 9.4% on travel allowances. Drugs and committee allowances accounted for 4.5% and 3.0% of expenditure, respectively.

**Figure 3:**
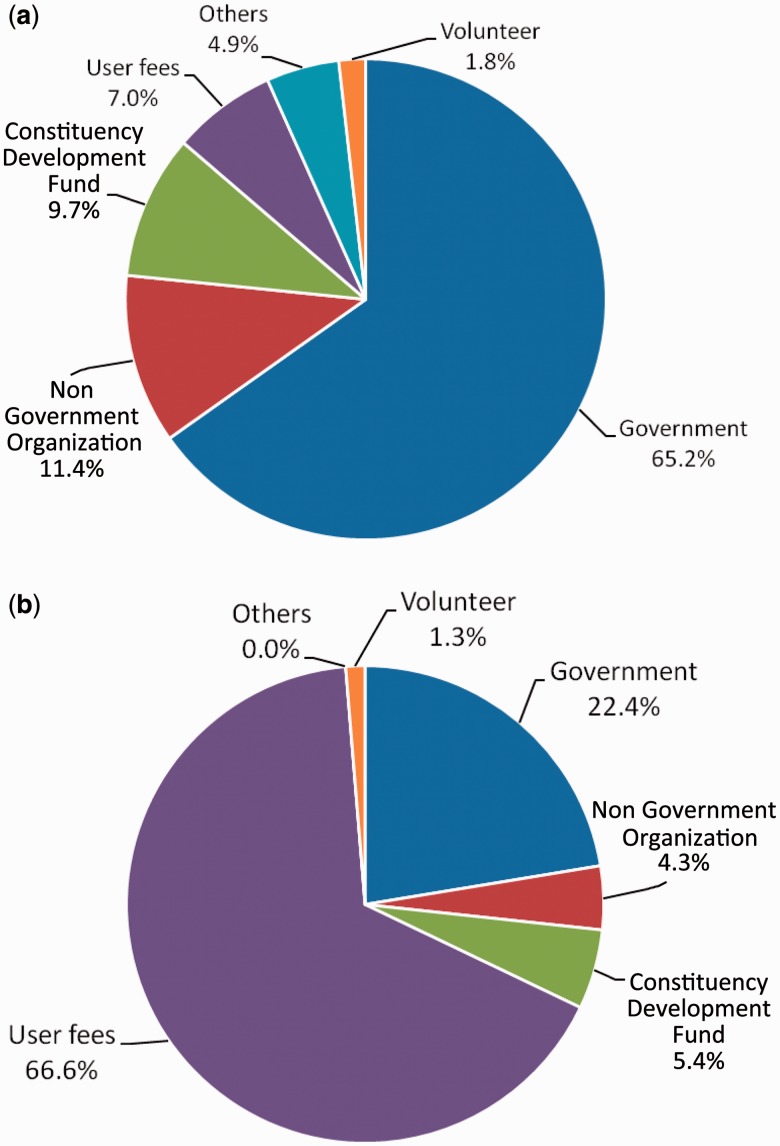
Source of salary for (a) qualified staff and (b) support staff. *Source:* In-charge interviews.

Since a substantial part of facility expenditure was on wages, we further investigated the sources of salary funding for facility staff. As shown in Figure 3a, professional employees were normally centrally employed, with almost two thirds (65.2%) of qualified staff being paid by the government, and only 7.0% receiving salaries from user fees. The picture was very different for support staff, two thirds of whom were paid through user fees (Figure 3b).

## Discussion

In this article we have presented nationally representative data from Kenya on user fee charges and revenues in health centres and dispensaries. The data were collected six years after the introduction of the 10/20 policy, which aimed to reduce previously high and variable user fees, and at a time of on-going discussion in the country on the complete removal of user fees from primary health care facilities. The study builds on earlier smaller scale studies which raised concerns about adherence to the 10/20 policy (Chuma et al. 2009; Opwora et al. 2010).

A limitation of this study is that data on facility adherence to user fee policy were based primarily on reports by in-charges, who may have under-reported fees charged. Although we also collected data on amounts paid by users during exit interviews, it is possible that our presence in the facility may have led to lower user fees being charged that day. However, if this were the case, then adherence data would have been even worse, supporting our overall finding of very frequent over-charging. Another limitation is the incompleteness of data on facility income and expenditure available from record reviews which meant that estimates could not be produced for nearly a quarter of facilities.

A key finding is that adherence to user fee policy across the country was very low. Moreover, a quarter of patients were required to purchase further items in private retail shops, so if all costs to patients were considered, adherence to the user fee policy would be even lower. Waivers on the basis of poverty were rare, granted on average for only 1% of outpatient curative visits.

Reasons for non-adherence to user fee policy have been documented in previous studies (Chuma et al. 2009; Opwora et al. 2010). Health workers were said to over-charge because other sources of revenue were insufficient to cover operational costs such as support staff and laboratory services, and because drug shortages were common, a practice in some settings endorsed by district officials. Moreover, exempted patients were argued to account for a high proportion of all patients, meaning that the revenue consequence of adherence were large. A further factor was that registration fees were paid before the patient was attended to by a health worker, making it unlikely that patients would be exempted for specific conditions diagnosed only after payment. Finally there was also confusion on the part of health workers, district officials and users on the policy details.

Studies nationally and internationally suggest that low adherence to user fee guidelines has potentially negative implications for access to health care, particularly for the poorest groups (Bitran and Giedion 2003; Chuma et al. 2009; James et al. 2006; Lagarde and Palmer 2008; Mbugua et al. 1995). For those who do access health care, these charges are likely to contribute to high cost burdens, particularly for poor households, and to expenditure levels that are potentially catastrophic (Chuma et al. 2007; Nabyonga et al. 2011; Onwujekwe et al. 2009; Xu et al. 2003). Indeed, it was these concerns that contributed to the introduction of the 10/20 policy in Kenya in 2004.

Furthermore, it has been noted that user charges often raise negligible revenue, frequently below 5% of total expenditure, and so health systems would experience little impact by removing them (Gilson 1997; Pearson 2004). In Kenya the average annual recurrent costs of health centres and dispensaries have been estimated at USD 114 000 and USD 39 000 respectively (author’s calculations based on the Kenya health sector costing model (Flessa et al. 2011), adjusted to 2010 prices). This implies that annual cash income would represent only approximately 1–2% of recurrent costs in dispensaries and 2–4% in health centres. However, our data also demonstrate that user fees can still play an important role in covering facility level costs, which are not provided from other sources. Nearly all cash income at facility level was generated from user fees, and, at least for rural facilities, almost all of this income was retained and used at the facility. This income was important in paying salaries for two thirds of support staff such as cleaners, patient attendants and security guards. These support staff have been reported by facility in-charges and committee members to be crucial in assisting with the day-to-day running of facilities, enhancing acceptability of services to patients by improving cleanliness and maintenance, and allowing health workers to concentrate more on the clinical duties that are essential to service provision (Opwora et al. 2010). Other important uses of user fees were non-drug supplies and equipment, travel allowances for staff and committee members, and stationery and photocopying, all of which potentially influence the motivation of staff and community volunteers, and the smooth functioning of facilities (Molyneux et al. 2007). The importance of user fee income at facility level has been similarly observed in other countries in terms of financing a proportion of staff income, supplementing pharmaceutical costs during stock outs, and covering other operating expenses (Nyonator and Kutzin 1999; Sepehri et al. 2005; Yates 2009).

Strengthening adherence to the 10/20 policy, and indeed any further reductions or removal of user fees in Kenya, will lead to income from user fees being lost, and this loss may need to be offset by other means if quality of care is not to be compromised (Gilson and McIntyre 2005; Hercot et al. 2011; McPake et al. 2011; Meessen et al. 2011a). Moreover, additional resources will be needed to cover any concomitant increase in utilization. Recognition of this need has led countries to compensate facilities in a number of ways, including providing more inputs such as drugs in kind, increasing staff salaries, reimbursing facilities per case, or introducing other variants of performance based pay (Meessen et al. 2011b). Common emerging concerns with these initiatives include financial sustainability and administrative burden.

In Kenya, the Health Sector Services Fund (HSSF) is one potential mechanism to compensate facilities for potential losses of user fee revenue. HSSF has been gradually rolled out nationally in public health facilities since 2010. The Government and development partners contribute to a central fund, which is used to credit funds directly into the bank accounts of approved facilities. HSSF funds are intended to cover the facility’s operational expenses (Government of Kenya 2009) while the Government continues to provide facility infrastructure, trained health workers, drug kits, and medical supplies directly to facilities. At the facility level, HSSF funds are managed by the Health Facility Committees which had previously been charged with management of user fee income. The HSSF funds at facility level can therefore potentially be used to offset the loss of income for facilities from a reduction in user fees charged. However, in the pilot of this mechanism in Coast Province, the increased funds did not lead to good adherence to user fee policy (Opwora et al. 2010). Reasons included lack of clarity to both providers and users on the details of the user fee policy, and a felt need for additional discretionary funds in many health facilities. Thus the new HSSF funds will potentially supplement rather than replace user fees collected. To achieve the expected interaction between HSSF and user fees, clear guidance should be given to facilities on user charges, and the adequacy of funding under HSSF should be reviewed. In addition, actual charging should be carefully monitored, both by managers and by strengthening community members’ awareness of their rights, and the possibility of making HSSF funding conditional on adherence to user fee policy should be considered.

## Conclusion

Adherence to the official user fee policy in Kenya’s public health centres and dispensaries was very low, with many patients paying for services that should have been free, others paying more than the specified amount, and few receiving waivers on the basis of poverty. Moreover, a quarter of patients were required to purchase additional supplies from private shops. No consistent patterns of association between facility characteristics and adherence were identified, with non-adherence common across facilities with a wide range of characteristics.

These findings raise serious concerns about the impact of user charges on access to essential health services and the financial burden, particularly for poorer households. However, the potential to enforce adherence to the user fee policy is likely to be heavily constrained by the facilities’ need for revenue, which gives them a powerful incentive to overcharge patients. At the time of the study user fees were the key source of cash income for facilities, covering items such as support staff, supplies and travel allowances, which are reported to be important in reducing workload for busy healthcare workers and ensuring the smooth running of the facility. Attempts to enhance adherence to the user fee policy and to further reduce official charges should be accompanied by strategies to compensate facilities for lost revenue. The Health Sector Services Fund provides a potential mechanism for this, but achieving the desired impact on user fee policy adherence will require the provision of adequate funds to each facility, and the close monitoring of fees charged.

## Footnotes

^1^ OANDA average exchange rate, 2010, USD1 = KES 76.18, www.oanda.com.

^2^ All districts were included in the sampling frame with the exception of Siaya and Kisumu West, both in Nyanza Province, and Kilifi in Coast Province because the intensity of Kenya Medical Research Institute research activities in these districts and Millennium Village activities in Siaya meant that the majority of health facilities in these districts were highly atypical.

^3^ Twenty-four (4.6%) of the facilities were excluded because they were not operational or were temporarily closed. If a facility was not operational at the time of the survey, we replaced the facility where possible with the next available facility on our list of randomly selected facilities.
